# A selective inhibition of c-Fos/activator protein-1 as a potential therapeutic target for intervertebral disc degeneration and associated pain

**DOI:** 10.1038/s41598-017-17289-y

**Published:** 2017-12-05

**Authors:** Hiroto Makino, Shoji Seki, Yasuhito Yahara, Shunichi Shiozawa, Yukihiko Aikawa, Hiraku Motomura, Makiko Nogami, Kenta Watanabe, Takeshi Sainoh, Hisakatsu Ito, Noriyuki Tsumaki, Yoshiharu Kawaguchi, Mitsuaki Yamazaki, Tomoatsu Kimura

**Affiliations:** 1 Department of Orthopaedic Surgery, Faculty of Medicine, University of Toyama, 2630 Sugitani, Toyama, 930-0194 Japan; 2 Department of Internal Medicine, Kyushu University Beppu Hospital, 4546 Tsurumihara, Tsurumiji, Beppu, Oita, 874-0838 Japan; 3 Toyama Chemical Co., Ltd., 4-1 Shimookui 2-chome, Toyama, 930-8508 Japan; 4 Department of Orthopaedic Surgery, Sainou Hospital, 70 Takata, Toyama, 930-0866 Japan; 5 Department of Anesthesiology, Faculty of Medicine, University of Toyama, 2630 Sugitani, Toyama, 930-0194 Japan; 6 Center for iPS Cell Research and Application, Kyoto University, 53 Kawahara-cho, Shogoin, Sakyo-ku, Kyoto, 606-8507 Japan

## Abstract

Intervertebral disc (IVD) degeneration is a major cause of low back pain. The transcription factor c-Fos/Activator Protein-1 (AP-1) controls the expression of inflammatory cytokines and matrix metalloproteinases (MMPs) that contribute to the pathogenesis IVD degeneration. We investigated the effects of inhibition of c-Fos/AP-1 on IVD degeneration and associated pain. A selective inhibitor, T-5224, significantly suppressed the interleukin-1β-induced up-regulation of *Mmp-3, Mmp-13* and *Adamts-5* transcription in human nucleus pulposus cells and in a mouse explant culture model of IVD degeneration. We used a tail disc percutaneous needle puncture method to further assess the effects of oral administration of T-5224 on IVD degeneration. Analysis of disc height, T2-magnetic resonance imaging (MRI) findings, and histology revealed that IVD degeneration was significantly mitigated by T-5224. Further, oral administration of T-5224 ameliorated pain as indicated by the extended tail-flick latency in response to heat stimulation of rats with needle-puncture-induced IVD degeneration. These findings suggest that the inhibition of c-Fos/AP-1 prevents disc degeneration and its associated pain and that T-5224 may serve as a drug for the prevention of IVD degeneration.

## Introduction

Low back pain (LBP) is a major problem worldwide. Seventy percent of people experience LBP at least once^1,2^. Lumbar intervertebral disc (IVD) degeneration is attributable mainly to LBP^3^, called discogenic pain, which often leads to disc herniation presenting as sciatica. The onset of IVD degeneration typically occurs and progresses during the third decade^4,5^, and no effective therapies able to restore the degeneration have yet appeared. Consequently, various treatments have been attempted, such as spinal fusion^6,7^ and disc arthroplasty as well as medication and physical exercise^8^. More recent strategies include gene therapy using stem cells^9^, artificial disc transplantation^10^ and administration of inhibitors of catabolic factors such as matrix metalloproteinases (MMPs)^11^.

IVD degeneration affects the annulus fibrosus (AF), nucleus pulposus (NP) and cartilage endplate. The NP plays important roles in maintaining homeostasis by producing components of the extracellular matrix (ECM) that are indispensable to the physiological viscoelastic properties of the IVD, including a type II collagen and proteoglycans^12,13^. During ageing or degeneration, an imbalance between the production and destruction of the ECM may occur in the NP. For example, Mern *et al*.^14^ found that in humans the expression of catabolic factors and inflammatory factors is up-regulated, and ECM production is down-regulated. Evidence indicates that MMPs and ADAM metallopeptidase with thrombospondin type 1 motif (ADAMTS) significantly contribute to IVD degeneration^15–18^. Further, some studies show that diverse biological response modifiers that inhibit catabolic factors are useful for the treatment of IVD degeneration^19–23^. Numerous studies^24,25^ show that the mitogen-activated protein kinase (MAPK) pathway, which includes c-Fos regulation and functions upstream of MMPs and IL-1β, plays an important role in destruction of the ECM. The activity of the heterodimeric transcription factor AP-1, comprising members of the c-Fos and c-Jun families of transcription factors, is induced by signalling through the MAPK pathway to regulate the expression of MMPs. It does this by binding to the AP-1 recognition sites in the promoter regions of MMP family genes^26–29^. For example, deletion of the AP-1 site from the promoter region of *Mmp-3* decreases transcriptional activity^30^, and c-Fos activation is essential for the induction of MMP-13 in IL-1β-treated SW1353 cells^31^. Further, the AP-1 complex indirectly upregulates the expression of ADAMTS-5 in IL-1β-treated human chondrocytes^32^. Moreover, c-Fos is associated with the downregulation of Col2 in NP cells^24^. Inhibition of c-Fos that act upstream in the pathways that synthesize catabolic factors may therefore represent an ideal strategy for developing molecularly targeted therapies to treat IVD degeneration.

c-Fos is also known as a neuronal marker that is activated in primary sensory neurons in rats exposed to pain^33^, and regulates the transcription of genes encoding enkephalin and dynorphin that affect the sensory nervous system^34–36^. Further, suppression of c-Fos expression inhibits nociception in adult rats^37,38^.

The novel benzophenone derivative T-5224 was rationally designed to serve as a potential drug to inhibit transcription regulated by AP-1^39,40^. Specifically, T-5224 was designed using 3D pharmacophore modeling based on the crystal structure of the AP-1-DNA complex^39,40^. It was subsequently shown to inhibit the activity of the c-Fos/c-Jun AP-1 heterodimer and to ameliorate rheumatoid arthritis in a mouse model^41^.

We hypothesized that inhibiting the expression and activity of c-Fos suppresses IVD degeneration and increases the pain threshold. Here, we used nucleus pulposus cell culture and rodent *ex vivo* and *in vivo* models of IVD degeneration to provide support for this hypothesis.

## Results

### Effect of a selective inhibitor of c-Fos/AP-1 on gene expression induced by interleukin (IL)-1β in human NP cells. A selective inhibitor

**Figure 1:**
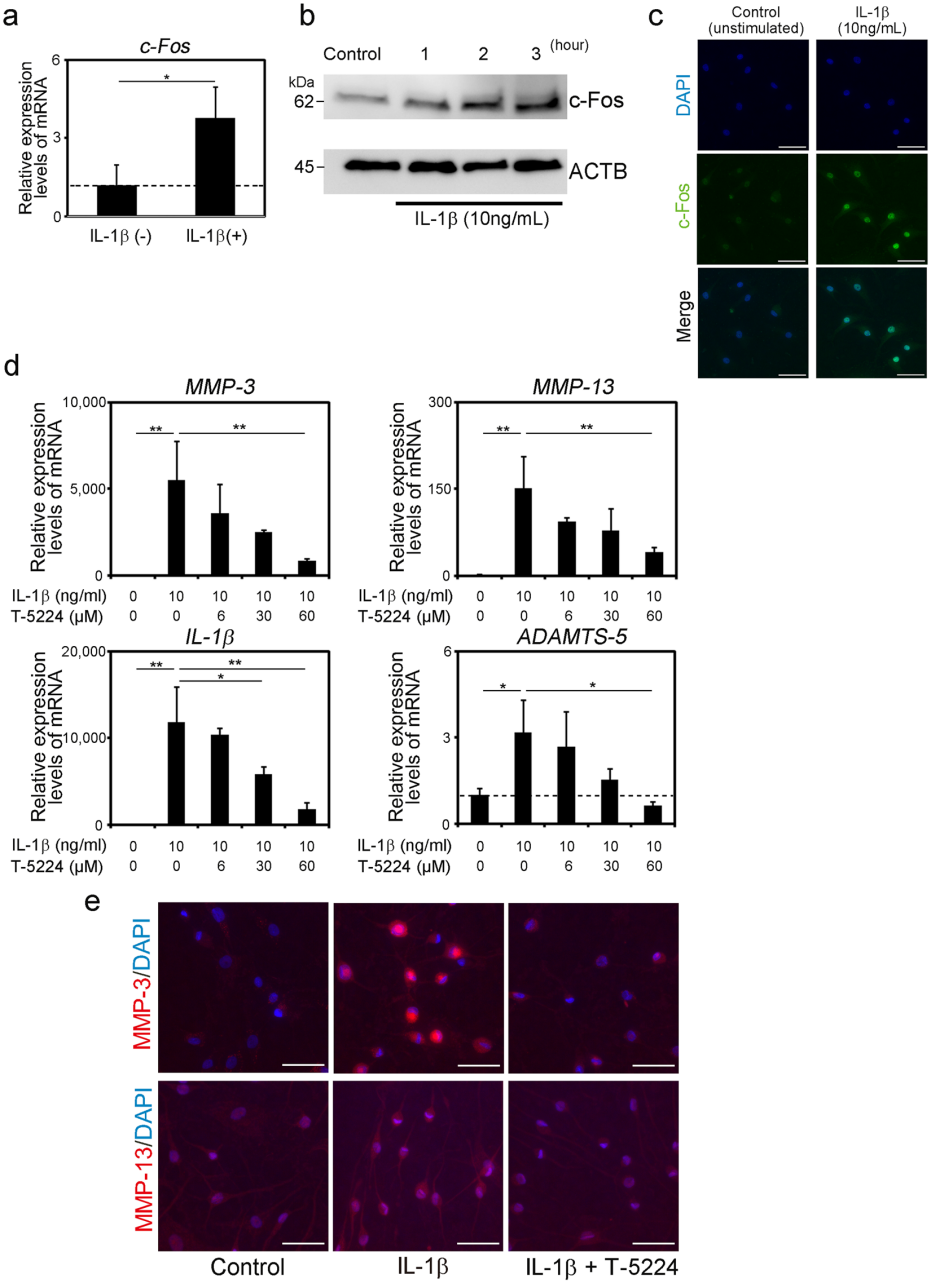
Effects of T-5224 on human NP cells treated with IL-1β. (**a**) Quantification of *c-Fos* mRNA in human nucleus pulposus (NP) cells 24 h after stimulation with or without IL-1β. (**b**) Western blot analysis of c-Fos expression. (**c**) Immunofluorescence staining for human NP cells 2 h after the stimulation with or without IL-1β. Scale bars = 50 μm. (**d**) Expression of genes encoding proteins involved in catabolism, and inflammation in human NP cells (n = 3) 24 h after stimulation with recombinant IL-1β with or without T-5224. Error bars denote the mean ± SD. *P < 0.05 and **P < 0.01 (Tukey–Kramer post-hoc test). (**e**) Immunofluorescence staining for IL-1β-induced human NP cells 24 h after treatment with T-5224 (60 μM). Scale bars = 50 μm.

T-5224, was newly generated from a cyclic disulfide decapeptide designed by 3D pharmacophore modeling based on the X-ray crystal structure of the basic region–leucine zipper domain of the AP-1–DNA complex^40,41^. IL-1β treatment of human NP cells significantly increased the expression of *c-Fos* mRNA in human NP cells, and the levels of c-Fos in the nucleus increased with time (Fig. 1a–c). Further, the levels of *MMP-3, MMP-13, ADAMTS-5* and *IL-1*β mRNAs were significantly increased by IL-1β treatment and significantly decreased by T-5224 in a dose-dependent manner (Fig. 1d). Inhibition of the IL-1β induced up-regulation of MMP-3 and -13 by T-5224 was apparent in immunofluorescence staining (Fig. 1e).

### Effects of T5224 on IL-1β induced expression of MMPs, Adamts5 and IL-1β in disc explant culture

**Figure 2:**
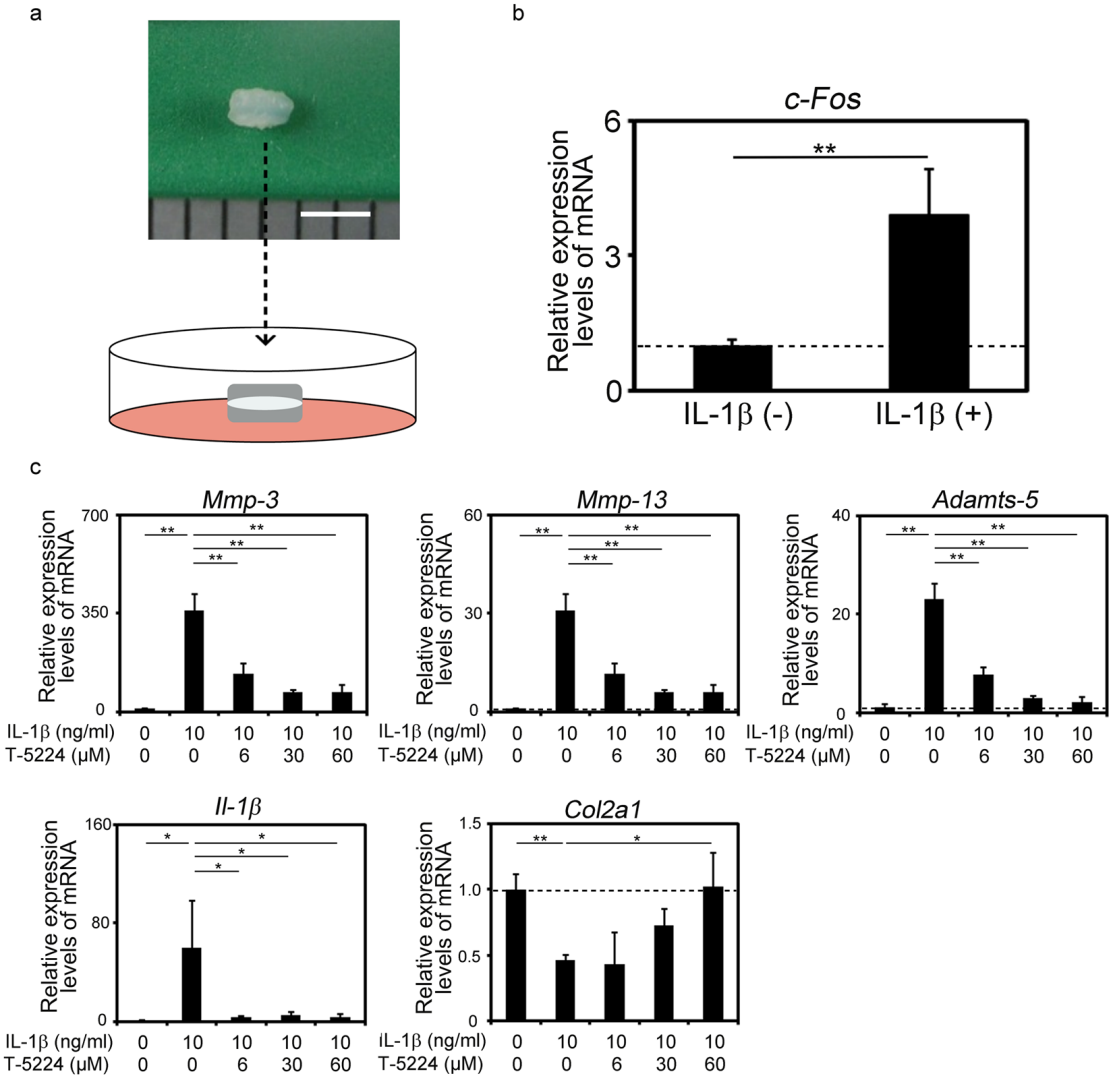
Effects of T5224 on IL-1β induced expression of MMPs, Adamts5 and IL-1β in disc explant culture. (**a**) Explant culture of mouse intervertebral discs (IVDs). Scale bar indicates 2 mm. (**b**) Expression of *c-Fos* in explant-cultured mouse IVDs (n = 3) treated with recombinant IL-1β. Error bars denote the mean ± SD. **P < 0.01 (Student *t* test). (**c**) Expression of genes encoding proteins involved in catabolism, ECM synthesis, and inflammation in explant-cultured mouse IVDs (n = 3) treated with recombinant IL-1β with or without T-5224. Error bars denote the mean ± SD. *P < 0.05 and **P < 0.01 (Tukey–Kramer post hoc test).

Next, we investigated the effects of T-5224 on mouse intervertebral disc degeneration induced by IL-1β in an *ex vivo* explant culture model (Fig. 2a). Recombinant IL-1β treatment increased the expression of *Mmp-3, Mmp-13, Adamts-5* and *Il-1*β in concert with the up-regulation of c-*Fos* and suppressed the expression of *Col2a1* (Fig. 2b,c). T-5224 significantly inhibited these effects of IL-1β in a dose-dependent manner (Fig. 2c). Further, the expression of *Col2a1* was significantly up-regulated by T-5524 (Fig. 2c), whereas the levels of *Adamts-4* and aggrecan (*Acan*) mRNAs were not significantly changed (data not shown).

**Figure 3:**
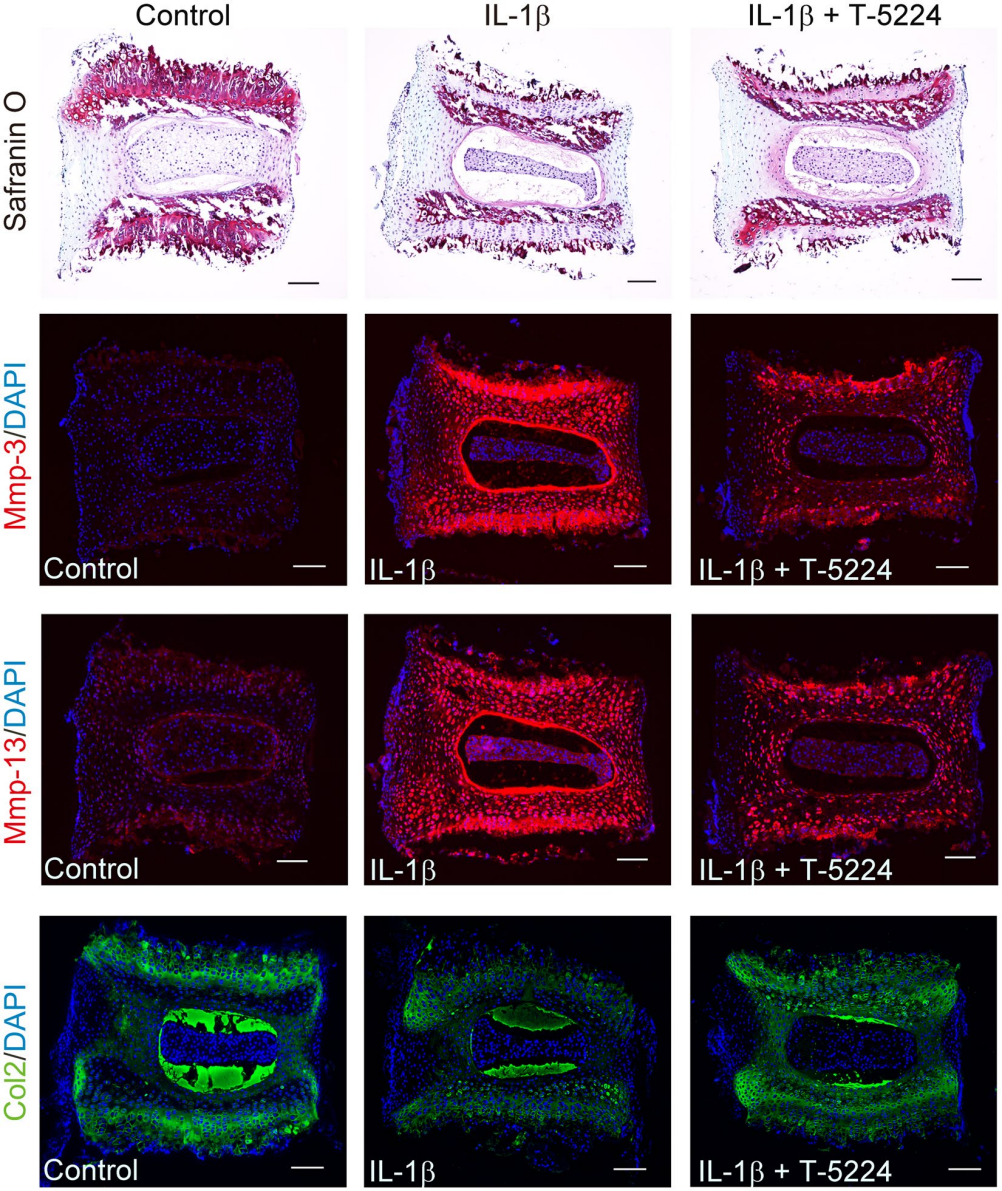
Histological analysis of explant-cultured mouse IVDs. Mouse IVDs were explant-cultured for three days with recombinant IL-1β (10 ng/ml) with or without T-5224 (50 μM). Paraffin-embedded sections were stained with safranin O-fast green-iron haematoxylin and subjected to immunohistochemical analysis using antibodies specific for Mmp-3, Mmp-13 and Col2. Scale bars = 100 μm.

IL-1β treatment induced reduced metachromasia by safranin O staining of the cartilage endplate and the inner layer of the AF and the shrinkage of the NP, which were improved by T-5224 treatment (Fig. 3). Further, T-5224 inhibited the IL-1β -induced up-regulation of Mmps-3 and -13 in the cartilage endplate and inner layer of the AF (Fig. 3). IL-1β treatment decreased the expression of type II collagen, which was recovered by T-5224 treatment in the inner and outer layers of the AF and cartilage endplate (Fig. 3).

### Effect of T-5224 on IVD degeneration *in vivo*

**Figure 4:**
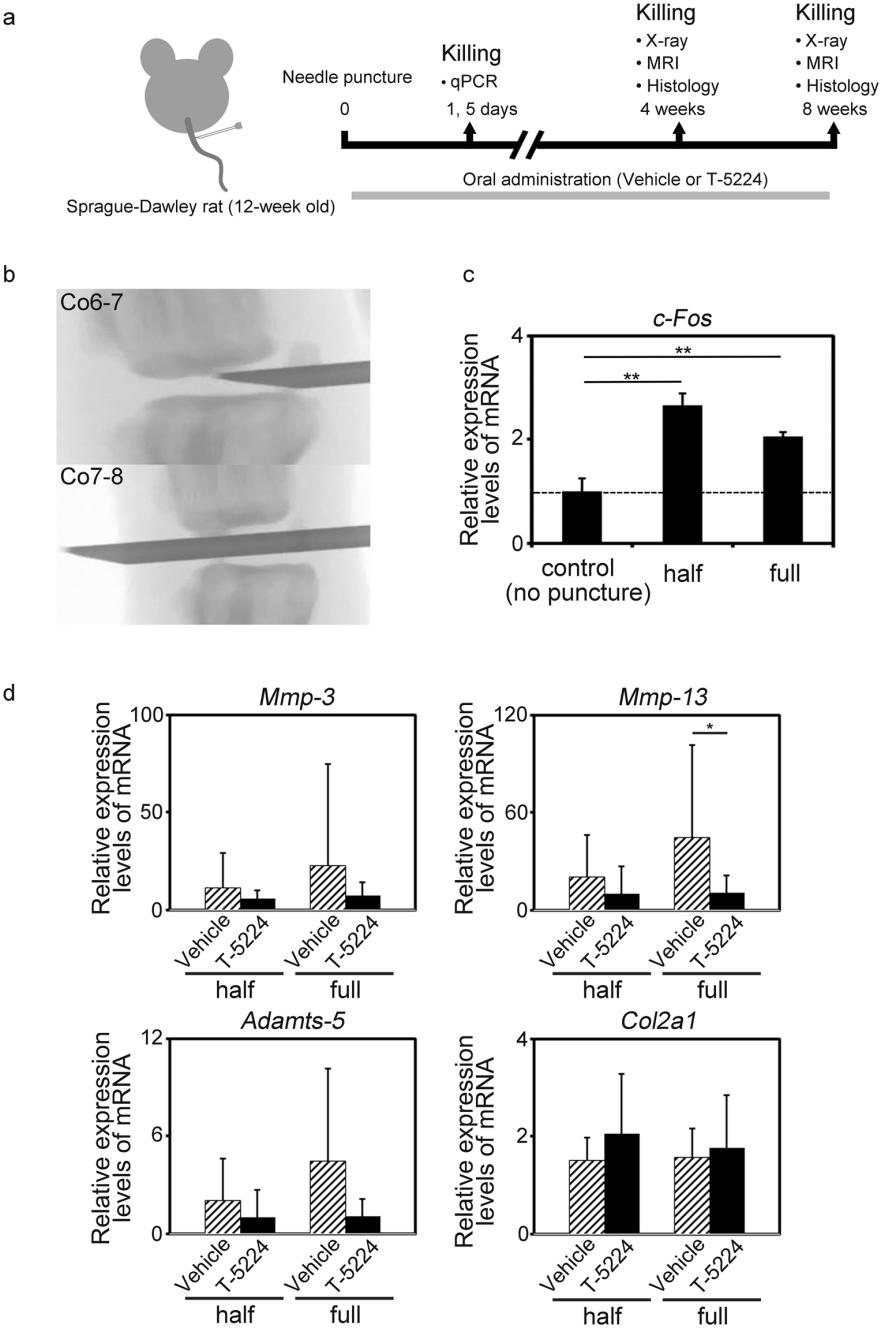
IVD degeneration induced by tail disc percutaneous needle puncture. (**a**) 12-week-old Sprague-Dawley rats underwent tail puncture and were killed at intervals for qPCR or image analysis. (**b**) Needle puncture methods: Method I, half puncture (Co 6-7); and Method II, full puncture (Co 7-8). (**c**) Expression of *c-Fos* in rat IVDs 24 h after needle puncture (n = 3). Error bars denote the mean ± SD. **P < 0.01 (Tukey–Kramer post hoc test. (**d**) Expression of *Mmp-3*, *Mmp-13*, *Adamts-5* and *Col2a1* mRNAs in rat IVDs 5 days after needle puncture (total n = 32; n = 16 for both vehicle and T-5224 groups). Error bars denote the mean ± SD. *P < 0.05 Student *t* test).

To analyze the effects of T-5224 on different aspects of IVD degeneration, we punctured the tails of 12-week-old rats to produce two types of IVD degeneration as follows: half puncture of coccygeal (Co) vertebrae Co6/7 and full puncture of Co7/8 (Fig. 4b). Co5-6 was not punctured and was used as the control. The rats were subsequently killed at different times, and the tissues were analyzed using quantitative real-time polymerase chain reaction (qPCR) or imaging techniques (Fig. 4a).

Needle puncture significantly increased the levels of *c-Fos* mRNA (Fig. 4c). The expression of *Mmp-3* and *Mmp-13* significantly increased in the degenerated IVD induced by half- or full-puncture (data not shown), and T-5224 treatment significantly suppressed the expression of *Mmp-13* in the IVD. The levels of *Mmp-3* and *Adamts-5* mRNAs were decreased in the T-5224 group compared with those of the vehicle group, although the differences were not statistically significant. The expression of *Col2a1* was not significantly altered in the degenerated IVDs and was not significantly up-regulated by T-5224 treatment (Fig. 4d).

**Figure 5:**
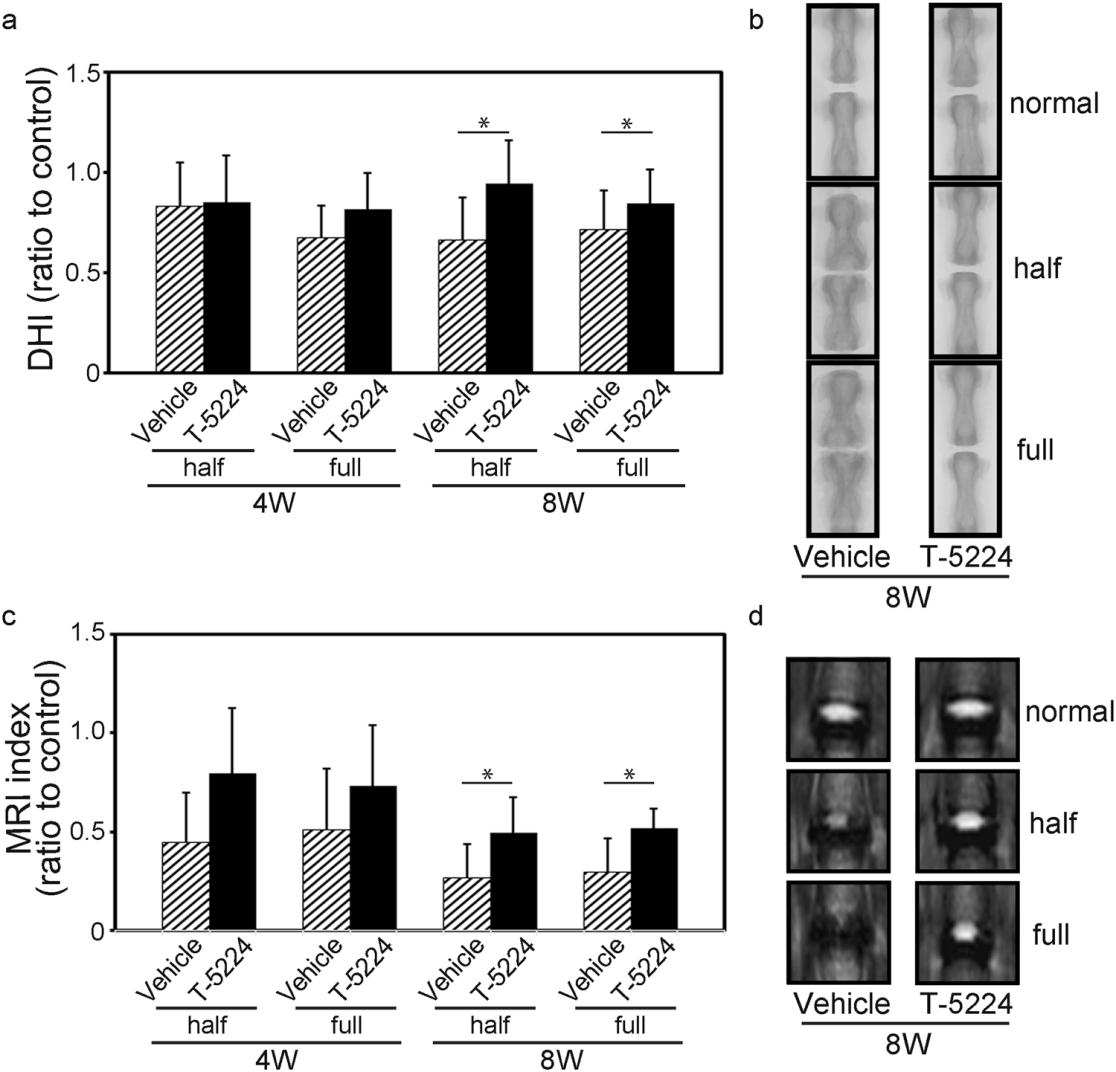
Effect of T-5224 on intervertebral disc degeneration. (**a**) X-ray image analysis of the disc height index (DHI) normalized to the control DHI (Co5/6 level) of rats with punctured discs administered vehicle solution or T-5224 (total n = 32; n = 7 for both vehicle and T-5224 groups at 4 weeks, n = 9 for both vehicle and T-5224 groups at 8 weeks). Error bars denote the mean ± SD. *P < 0.05 (Student *t* test). (**b**) Representative X-ray coronal image of the coccygeal spine of rats treated for 8 weeks with vehicle solution or T-5224. (**c**) MRI index normalized to the mean T2 value of the control level (Co5-6) of punctured discs of rats administered vehicle solution or T-5224 (total n = 32; n = 7 for both vehicle and T-5224 groups at 4 weeks, n = 9 for both vehicle and T-5224 groups at 8 weeks). Error bars denote the mean ± SD. *P < 0.05 (Student *t* test). (**d**) Representative T2-mapping image of coccygeal IVDs of rats treated for 8 weeks with vehicle solution or T-5224.

X-ray imaging did not reveal significant differences between animals treated for 4 weeks with vehicle or T-5224 after they were subjected to either puncture method. Eight weeks after the administration of either type of needle puncture, the disc height index (DHI) was significantly higher in the T-5224 group compared with that of the vehicle group (Fig. 5a,b). In vehicle treated rats, the formation of osteochondrophytes adjacent to the vertebral body, widening of the endplate and a reduction in the longitudinal length of the vertebra were observed (Fig. 5b). The DHI of rats subjected to the full puncture method was less compared with that of those subjected to half-puncture method during weeks 4 and 8, although the difference was not significant.

The MRI index was not significantly different between the vehicle and T-5224 groups on treatment at four weeks. After 8 weeks, however, the T-5224 group maintained significantly higher T2-weighted MRI signal intensity compared with the vehicle group, suggesting inhibition of the progression of IVD degeneration (Fig. 5c,d).

**Figure 6:**
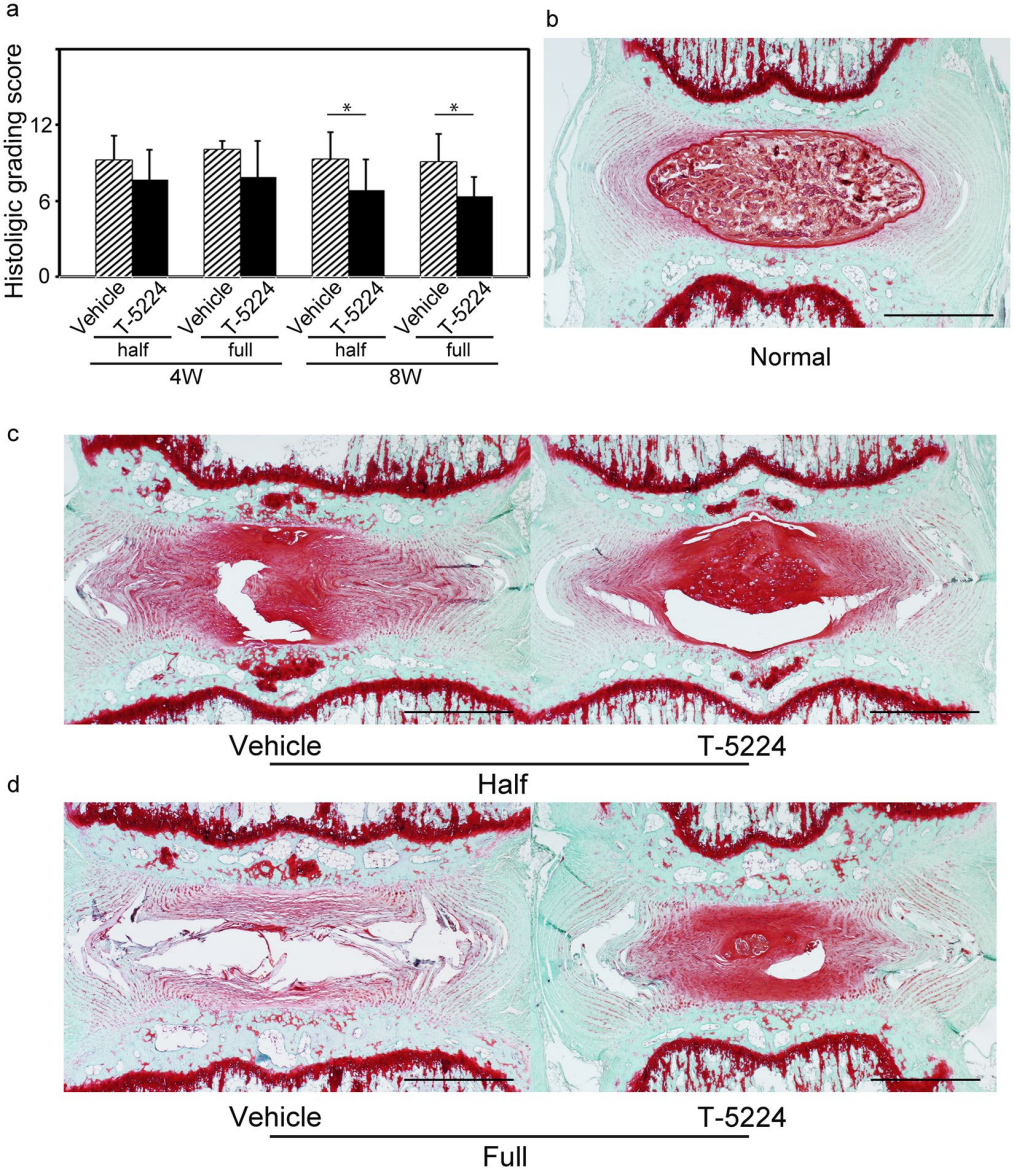
Effects of T-5224 on IVD degeneration of tail-punctured rats. (**a**) Histological grading scores of punctured discs of rats administered vehicle solution or T-5224 (total n = 32; n = 7 for both vehicle and T-5224 groups at 4 weeks, n = 9 for both vehicle and T-5224 groups at 8 weeks). Error bars denote the mean ± SD. *P < 0.05 (Student *t* test). (**b**) Safranin O staining image of a control disc. (**c**) Safranin O staining of half-punctured discs 8 weeks after the administration of vehicle solution or T-5224. (**d**) Safranin O staining of a full-punctured disc 8 weeks after the administration of vehicle solution or T-5224. Scale bars = 1 mm.

The histological grade of the T-5224 group was significantly better than that of the vehicle group at 8 weeks (Fig. 6a). The administration of T-5224 significantly affected the histochemical changes associated with IVD degeneration. Eight weeks after treatment with vehicle or T-5224 treatment, the vehicle group lost NP tissues, which were replaced by a fibrocartilaginous tissue compared with the normal disc (Fig. 6b–d). The severely degenerated discs of the vehicle treatment group lost proteoglycans and exhibited collapsed and wavy fibrocartilage lamellae typical of the AF (Fig. 6c,d). In discs treated with T-5224, safranin-O staining demonstrated the maintenance of the IVD structure comprising a lightly stained fibrocartilage lamellae and NP (Fig. 6c,d). The intraclass correlation coefficient (ICC) of inter-observer reliability was 0.89 (95% confidence interval, 0.78–0.98), indicating close agreement. T-5224 inhibited the expression of Mmp-13 in the cartilage endplate and NP compared to vehicle at 8 weeks after puncture (Supplementary Fig. 2).

### Effect of T-5224 on pain associated with IVD degeneration using the tail disc needle puncture method

**Figure 7:**
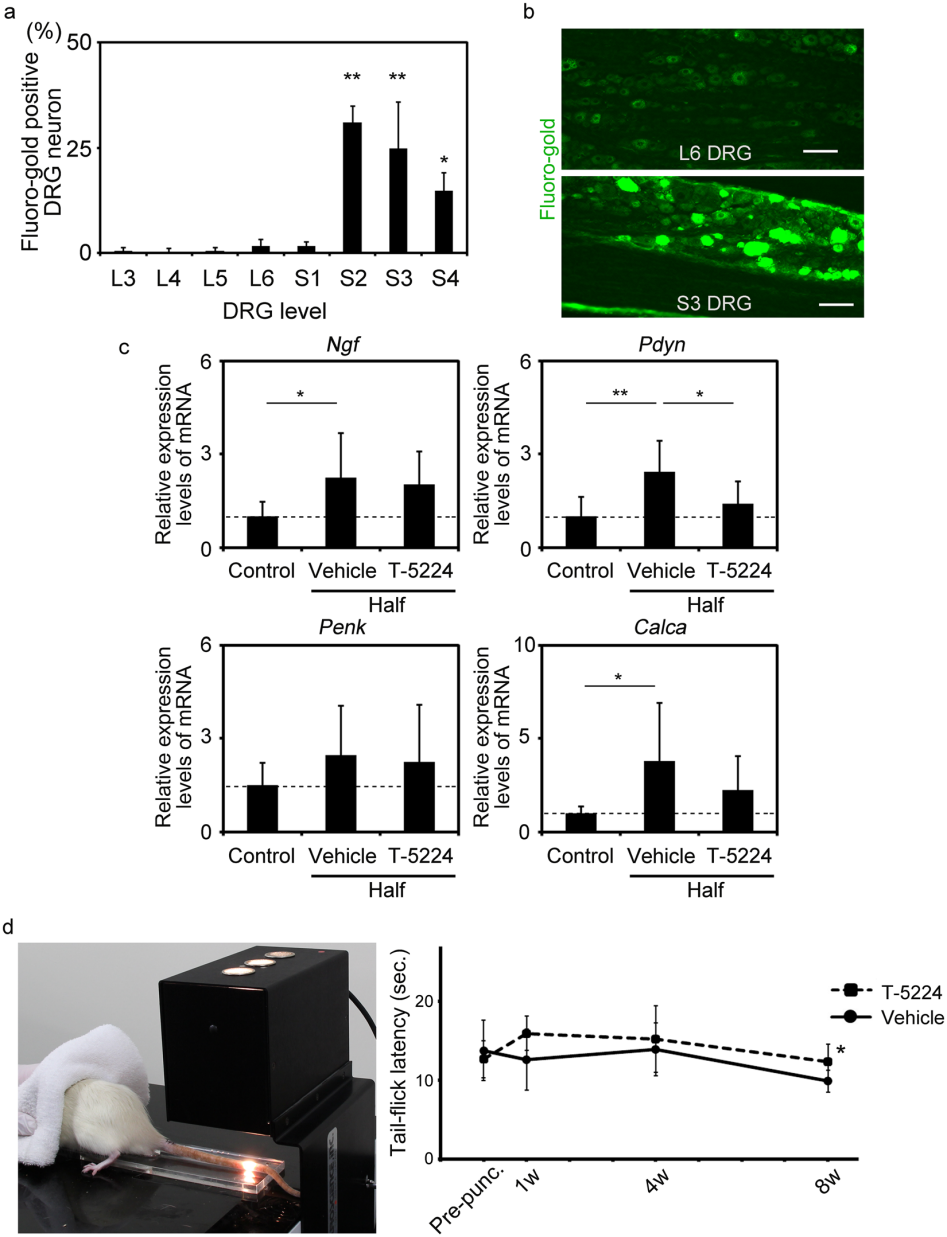
Effect of T-5224 on pain associated with IVD degeneration. (**a**) Retrograde fluoro-gold (FG) labelling of dorsal root ganglia (DRG) neurons innervating the Co6-7 intervertebral disc (n = 3). Error bars denote the mean ± SD. *P < 0.05 and **P < 0.01 (Tukey–Kramer post-hoc test). (**b**) Representative image of FG-labeled DRG neurons. (**c**) Expression of mRNAs encoding nerve growth factor (*Ngf*), prodynorphin (*Pdyn*), proenkephalin (*Penk*) and calcitonin/calcitonin-related polypeptide, alpha (*Calca*) in DRG neurons one week after Co6-7 puncture (total n = 9; n = 3, 9 level DRG neurons [S2–4] for all groups). (**d**) Effect of T-5224 on pain in the tail-flick test. Left image shows the tail-flick method (total n = 16; n = 8 for both vehicle and T-5224 groups). Error bars indicate the mean ± SD. *P < 0.05 (Student *t* test).

Retrograde fluoro-gold (FG) labelling of the Co6-7 IVD revealed significant numbers of FG-labeled dorsal root ganglia (DRG) neurons in the S2–S4 layers of the DRG compared with those of the others (Fig. 7a,b). This result indicates that the S2–S4 DRG innervates Co6-7 of the IVD (Fig. 7b). Therefore, S2-4 DRG neurons were used for subsequent analyses.

The levels of nerve growth factor (*Ngf*), prodynorphin (*Pdyn*) and calcitonin/calcitonin-related polypeptide, alpha (*Calca*) mRNAs in S2–S4 DRG neurons significantly increased one week after puncture to IVD Co6-7. In contrast, the expression of proenkephalin (*Penk*) was not significantly altered (Fig. 7c). T-5224 treatment significantly suppressed the expression of *Pdyn* in S2–S4 DRG neurons 1 week after puncture compared with treatment with the vehicle. The levels of *Ngf and Calca* mRNAs were not significantly changed by T-5224 treatment (Fig. 7c). Next, we conducted microarray analysis to determine whether genes other than *Pdyn* were involved in pain associated with IVD degeneration. We found that the expression of *Pdyn* in the DRG was significantly downregulated by T-5224 compared with vehicle (Log FC = −2.92, P = 0.02). Gene ontology (GO) analysis revealed that down-regulated genes in DRG neurons of T-5224-treated rats were associated with opioid and neuropeptide (Supplementary Table 4). These finding suggests that down-regulation of *Pdyn* expression may represent a major mechanism of the regulation of the expression of genes that are associated with pain during T-5224 treatment of the punctured intervertebral disc.

Tail-flick latency, which we measured as a surrogate of pain, was shortened with time after puncture of the vehicle and T-5224 treatment groups. The latency of the T-5224 treatment group was significantly longer 8 weeks after puncture compared with that of the vehicle treatment group (Fig. 7d).

## Discussion

**Figure 8:**
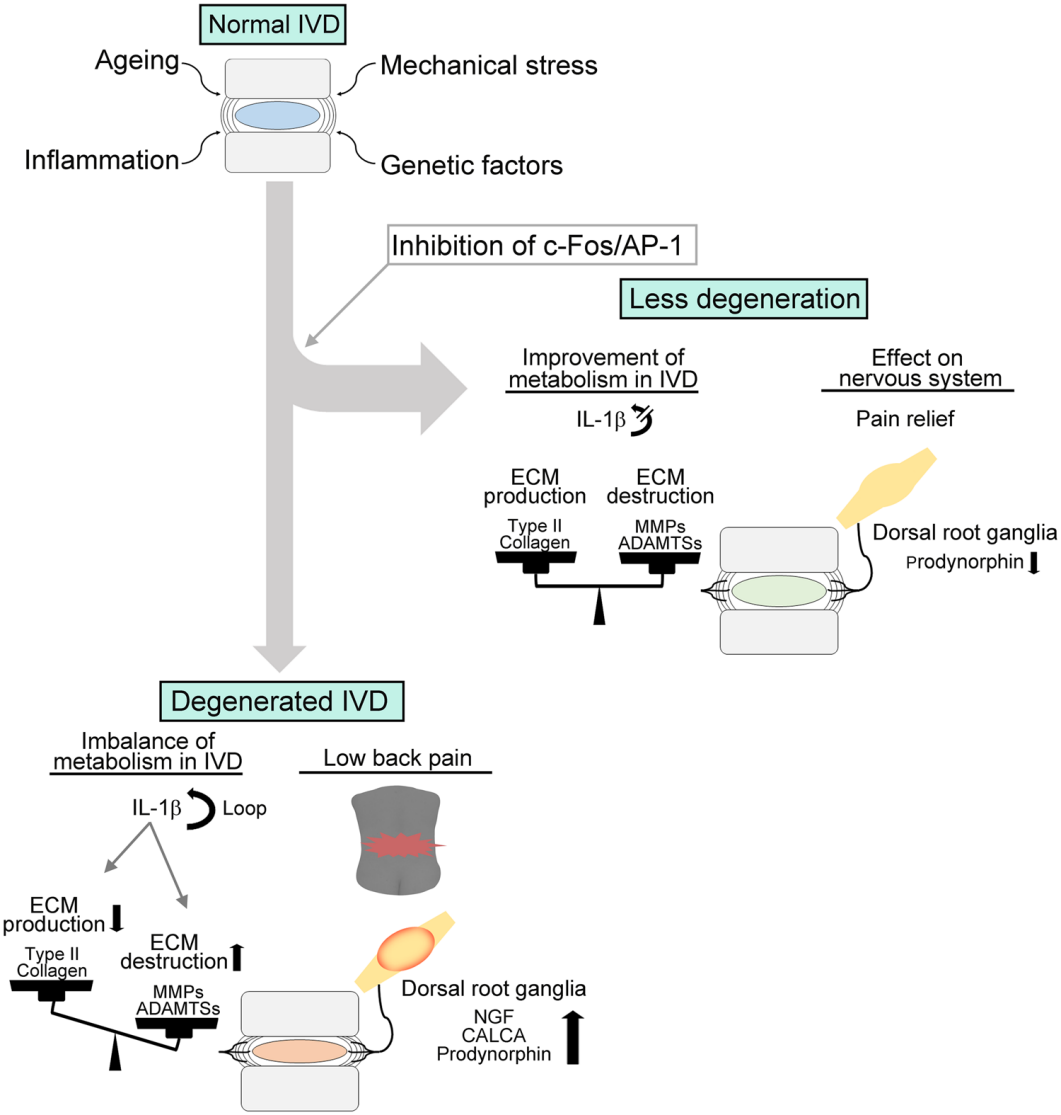
Proposed mechanism of action of the c-Fos/AP-1 inhibitor. Normal IVDs undergo degenerative change caused by factors such as aging, mechanical stress, genetic predisposition or inflammation. Subsequently, pain is induced by the degenerated IVD. Administration of a c-Fos/AP-1 inhibitor should lead to restoration of the degenerated IVD and amelioration of the associated pain.

We show here that the selective c-Fos/AP-1 inhibitor T-5224 prevented IVD degeneration in *ex vivo* (explant culture) and *in vivo* (tail puncture) rodent models of IVD degeneration. In the mouse explant culture model, IL-1β treatment increased the expression of *Mmp* and *Adamts* family members. These metalloproteinases degrade the ECM in the IVD via an increase in the expression of *c-Fos* and decreased expression of the ECM components *Col2a1*. Image analysis of rats subjected to the needle puncture technique revealed that oral administration of T-5224 preserved disc height on X-ray and T2 signal intensity of MRI, and histological analysis indicated that the progression of IVD degeneration, which was associated with significantly increased expression of *c-Fos* mRNA, was inhibited. Regarding as disc height on X-ray, a reduction in the longitudinal length of the vertebra were observed in the vehicle-treated rats. This phenomenon in the vehicle-treated rats should cause underestimation of the difference in the DHI shown in Fig. 5a. Further, T-5224 reduced hyperalgesia induced by heating of the tail. Figure 8 presents a model depicting the effects of T-5224. Together, these findings show that T-5224 has the potential for translation to the clinic as an orally administered drug that suppresses IVD degeneration and its associated pain. To our knowledge, no such is available for the treatment of patients with LBP.

T-5224 inhibits a broad range of factors that function downstream of AP-1 and suppresses the expression of IL-1β, which is a key factor that contributes to IVD degeneration. The cytokine IL-1β, which is a major regulator of inflammation, induces the expression of MMPs and ADAMTS family members that are associated with IVD degeneration^42^. IL-1β increases its own expression through a positive feedback loop^43^ requiring activation of the MAPK/AP-1 pathway^44^. Further, the up-regulation of endogenous IL-1β expression induced by exogenously added recombinant IL-1β is suppressed by T-5224. Consequently, T-5224 may interrupt the positive feedback loop that mediates IVD degeneration via the IL-1β/MAPK/AP-1 pathway.

In the present study, we orally administered T-5224 to treat IVD degeneration in a rat model system. Although drugs used to treat IVD degeneration are generally given by local administration^45^, some studies have employed oral administration^11,46^. Suzuki *et al*. found that the oral administration of N-acetyl cysteine suppresses the expression of tumor necrosis factor-alpha and MMP-3 subsequent to the induction of oxidative stress in a rat needle-puncture model^11^. Moreover, oral administration of test compounds is rational, because the degenerative changes of IVDs are observed at multiple levels^4,5^. Further, IVD degeneration is a potential cause of LBP, although it is difficult to determine the level of the causative disc, even using methods such as discography and disco-block^47^. Therefore, we suggest that oral administration may improve treatment of IVD degeneration.

In contrast, oral administration raises concerns about whether the drugs reach the target IVD and whether systemic adverse side effects may occur. The disc is virtually avascular, and neighbouring blood vessels contribute to its supply of nutrients and drugs^48–51^. Perlewitz *et al*. reported that low molecular contrast media such as gadopentetate (molecular weight 546) injected intravenously readily diffuse into the IVD compared with high molecular compounds such as gadolinium-polylysine (molecular weight 40,000)^52^. Given that the molecular weight of T-5224 is approximately 517, its diffusion into the IVD is likely. Treatment with T-5224 *in vivo* significantly decreased the expression of *Mmp-13*, in contrast to vehicle treatment, and the expression of other catabolic factors such as *Mmp-3* and *Adamts-5* was reduced as well. These findings support the conclusion that T-5224 reached the IVD.

Inhibition of cell signalling pathways or matrix enzyme activity may cause adverse side effects. For example, a broad-spectrum MMP inhibitor causes musculoskeletal side effects^53^. To our knowledge, there are no published studies of the side effects of c-Fos/AP-1 inhibitors on human or rodents. c-Fos knockout mice suffer osteopetrosis^54^, and c-Fos is essential for the differentiation of osteoclasts^55^. Therefore, T-5224 may influence bone metabolism, particularly bone resorption. T-5224 was developed for the treatment of rheumatoid arthritis (RA), and a clinical trial found that oral administration had no serious side effects in phase II trial. In the present study, T-5224-treated rats did not exhibit apparent side effects.

The MAPK pathway plays an important role in pain^56^, and c-Fos is expressed in the DRG and the posterior horn of spinal cord after exposure to noxious stimuli^33,57–59^. In addition, Pdyn has been reported to be associated with the development and maintenance of hypersensitivity and the MAPK/c-Fos pathway^57,60–62^. In the present study, T-5224 reduced hyperalgesia caused by thermal stimulation of the tail 8 weeks after rats were subjected to a coccygeal disc puncture. This may be caused by the inhibition of the c-Fos/AP-1 pathway in the tissues of the nervous system, such as the DRG. Moreover, expression of *Pdyn* in the DRG was suppressed by administration of T-5224. However, the inhibitory effect of T-5224 on disc degeneration may influence pain-related behaviour, which will be a focus of our future studies.

In conclusion, the selective c-Fos/AP-1 inhibitor T-5224 prevented disc degeneration in an explant culture model of mouse IVD induced by IL-1β and in a rat model of IVD degeneration induced by needle puncture. We conclude that the effects of T-5224 were mediated via the suppression of catabolic factors induced by c-Fos/AP-1. T-5224 treatment also significantly suppressed the expression of *Pdyn* in DRG neurons, and oral administration of T-5224 extended the tail flick latency as a measure of heat stimulation in the rat tail-puncture model. These findings suggest that T-5224 may exert an anti-hyperalgesic effect via MAPK/c-Fos signalling that induces the expression of PDYN. It is important to note that T-5224 is safe for oral administration to humans. Therefore, the selective c-Fos/AP-1 inhibitor T-5224 appears suitable for development as a therapeutic agent for the treatment of IVD degeneration in humans.

## Methods

### Ethics statement

The experiments conducted on human tissue was approved by the Ethical Review Board of University of Toyama (No. 28–108). All animal experiments were approved by the Animal Experiment Committee of the University of Toyama, Japan (A2015MED-1). All methods were performed in accordance with the relevant laboratory guidelines and regulations.

### Reagents

T-5224 (c-Fos/AP-1 inhibitor), 3-{5-[4-(Cyclopentyloxy)-2-hydroxybenzoyl]-2-[(3-hydroxy-1, 2-benzisoxazol-6-yl)methoxy]phenyl}propionic acid) was provided by Toyama Chemical Co., Ltd (Fig. 1a). T-5224 was dissolved in polyvinylpyrrolidone (PVP) solution (vehicle) for oral use. T-5224 was dissolved in DMSO and added to the culture for the *in vitro*.

### Animals

We used Sprague-Dawley rats and C57BL/6 mice. All experiments were performed following the criteria of the Guide for the Care and Use of Laboratory Animals^63^. Rats and mice were kept two per cage under standard conditions with a 12/12-hour light/dark cycle and free access to food and water.

### Isolation of human NP cells

Human NP tissue was obtained during surgery for scoliosis after receiving the patient’s informed consent. Tissue samples were digested at 37 °C overnight. After digestion, the isolated NP cells were cultured as a monolayer in DMEM containing 10% foetal bovine serum. Low-passage cells (passage 2) were used for all experiments. When the cells were 80% confluent, they were cultured in serum-free DMEM for 12 h and then treated with 10 ng/ml IL-1β with different concentrations of T-5224 or the vehicle.

### Mouse IVD explant culture

Lumbar IVDs were harvested from 2-week-old mice and cultured as previously described^64^ in 500 µL α-modified essential medium. IVDs were treated with 10 ng/ml mouse IL-1β (R&D Systems, Minneapolis, MI, USA) for 24 h with varying concentrations of T-5224.

### Tail-puncture model of IVD degeneration

Surgery was performed as previously described^65^. Briefly, rats were anesthetized with an injection of 0.15 mg/kg medetomidine, 2 mg/kg midazolam and 2.5 mg/kg butorphanol tartrate. We then used a sterile 20-gauge needle to puncture the tail from the dorsal to the ventral side. Two puncture methods were used as follows: I. half-penetration puncture (approximately 5 mm from the skin) and II. full-penetration puncture from the ventral to dorsal skin through the center of the NP (Fig. 4b). Co5-6 was not punctured and used as the control, Co6-7 and Co7-8 were punctured using Methods I and II, respectively. After recovery from anesthesia for 24 h, T-5224 (100 mg/kg each) or vehicle solution (polyvinylpyrrolidone, PVP) was administered once daily using a stainless steel feeding needle.

### qPCR

RNA was extracted using ISOGEN (Nippon Gene, Toyama, Japan), and 500 ng (mouse), 100 ng (rat) or 500 ng (human) total RNA was reverse-transcribed. Gene expression was quantified using qPCR with a Gene Amp 7000 Sequence Detection System (Applied Biosystems, Warrington, UK). The sequences of the primers are shown in Supplementary Table 1–3. Data were normalized to those of *GAPDH* mRNA, and relative gene expression of rat IVD was determined using ∆∆Ct method (ratio at Co5-6 for the tail-puncture data).

### Histological analysis

Samples of mouse and rat IVDs were fixed in 4% paraformaldehyde (PFA) and embedded in paraffin. Sections were stained with hematoxylin and eosin (HE) as well as with safranin-O. Disc degeneration in the tail-puncture method was quantified using a histological grading scale as previously described^66^. Grading was conducted by three orthopaedic surgeons who were uninformed of the nature of the experiment. We then calculated the intraclass correlation coefficient (ICC) to assess inter-observer reliability.

### Immunohistochemistry

Paraffin-embedded sections were deparaffinized and the antigen was retrieved. After blocking the sections with 10% normal goat serum, sections were incubated with the primary antibody at 4 °C overnight. The primary antibodies, sources, and dilutions were as follows: rabbit anti-Mmp3 (ab53015, Abcam, UK, 1:1000), rabbit anti-Mmp13 (ab39012, Abcam, UK, 1:1000), and mouse anti-type II collagen antibody (MS235P0, Thermo Scientific, MA, USA, 1:200). Secondary antibodies conjugated to Alexa Fluor (Life Technologies, MA, USA, 1:1000) or DAB was used to detect immune complexes, and DAPI (Dojindo Molecular Technologies, Kumamoto, Japan, 1:1000) was used to stain nuclei.

### Immunofluorescence staining

The human NP cells were plated in 4-well culture slide and incubated for 24 h. The cells were treated with 10 ng/ml IL-1β with or without T-5224, then fixed for 10 min with 4% paraformaldehyde, permeabilized with 0.5% Triton X-100 in PBS for 8 min, blocked with protein block (X0909, Agilent Technologies, USA) for 1 h, and incubated overnight at 4 °C with primary antibodies. The cells were washed and incubated with anti-rabbit Alexa Fluor and DAPI for 1 h at room temperature for nuclear staining.

### Western blotting

Nuclear extracts (10 μg) were subjected to SDS–PAGE, transferred to a polyvinylidene fluoride membrane that was blocked with 5% skim milk and then incubated with an anti-c-Fos antibody (sc-52, Santa Cruz Biotechnology, Inc., TX, USA, 1:1000) or an anti-ACTB antibody (#4970, Cell Signaling Technology, MA, USA, 1:1000). Full blotting images corresponding to the immunoblottings shown in the main figures are provided as Supplementary Fig. 1.

### Image analysis

X-ray imaging and MRI of the rat tail (Co5-6, Co6-7 and Co7-8) were performed. The intervertebral disc space shown on the X-ray images was evaluated using disc height index (DHI) as previously described^65,66^. Coronal T2 mapping images obtained using a Varian Unity Inova 4.7 T MRI were evaluated to determine disc degeneration. Regions of interest (ROI) after T2 mapping were defined using ImageJ^67^ as square areas, which represented the NP. After calculating the average T2 value of the ROI at each disc level, the values of Co6-7 and Co7-8 were recalculated using the Co5-6 (control) value as the reference (MRI index).

### Retrograde fluoro-gold (FG) neurotracer labelling of dorsal root ganglia (DRG) neurons innervating Co6-7 IVD

Retrograde FG labelling was performed as previous described^68^. Briefly, Sprague-Dawley rats were anesthetized and a dorsal longitudinal incision was made to expose the Co6-7 intervertebral disc. A 20-gauge needle with its tip filled with FG neurotracer (Fluorochrome, Denver, CO) was inserted into the center of the intervertebral disc. One week after surgery, bilateral DRG neurons from L3 to S4 were harvested and fixed in 4% PFA.

### Microarray and gene ontology (GO) analyses

Total RNA from the rat S2 DRG treated with T-5224 or vehicle (n = 3) was analysed. 20 ng of total RNA was labelled with a SureTag Complete DNA labelling Kit (Agilent Technologies, USA) and hybridized to the SurePrint G3 Rat GE v2 8 × 60 K Kit (Agilent Technologies, USA). The hybridized microarrays were scanned using an Agilent Microarray Scanner (Agilent Technologies, USA). Feature Extraction software (Agilent Technologies, USA) was used for analysing the data process. Genes differentially expressed between vehicle and T-5224 treated were statistically selected according to the following setting (p < 0.05, |LogFC| > 1). GO terms over-represented in genes differentially expressed compared to the full set of GO terms in rat from NCBI EntrezGene were analysed by Fisher’s exact test.

### Analysis of pain-related behaviour

The effect of T-5524 on pain was assessed using a tail flick test^69^. The tails of 12-week-old rats were punctured with a sterile 20-gauge needle from the dorsal to the ventral side. One week later, radiant heat was applied to the tail 5 cm from the tip using a tail-flick analgesia meter (IITC, Woodland Hills, CA, USA) with or without T-5224. Rats were calmed by enclosing their heads with a towel on the apparatus. The intensities of the radiant heat were adjusted so that the latencies of control rats were 10–14 s. To avoid tissue damage, the heat stimulus was discontinued after 25 s (cut-off latency).

### Statistical analysis

We used ANOVA followed by the Tukey-Kramer post-hoc test for the analyses, and P values < 0.05 were considered statistically significant. The Student *t* test was used to compare the results of X-ray imaging, MRI, histologic grading scores, and qPCR data between the vehicle and T-5224 groups in *in vivo* analyses.

## Electronic supplementary material

Supplementary information
