# Supplementary material for: A selective inhibition of c-Fos/activator protein-1 as a potential therapeutic target for intervertebral disc degeneration and associated pain
Source: Sci Rep. 2017 Dec 5;7:16983. doi: 10.1038/s41598-017-17289-y (PMC5717052; doi:10.1038/s41598-017-17289-y)
Supplement: Supplementary file 1 — Supplementary information [file 41598_2017_17289_MOESM1_ESM.doc]

**A selective inhibition of c-Fos/activator protein-1 as a potential therapeutic target for intervertebral disc degeneration and associated pain**

Hiroto Makino^1^, MD, Shoji Seki^1^, MD, PhD, Yasuhito Yahara^1^, MD, PhD, Shunichi Shiozawa^2^, MD, PhD, Yukihiko Aikawa^3^, PhD, Hiraku Motomura^1^, MD, PhD, Makiko Nogami^1^, MD, PhD, Kenta Watanabe^1^, MD, Takeshi Sainoh^4^, MD, PhD, Hisakatsu Ito^5^, MD, PhD, Noriyuki Tsumaki^6^, MD, PhD, Yoshiharu Kawaguchi^1^, MD, PhD, Mitsuaki Yamazaki^5^, MD, PhD, Tomoatsu Kimura^1^, MD, PhD

1) Department of Orthopaedic Surgery, Faculty of Medicine, University of Toyama, 2630 Sugitani, Toyama, 930-0194, Japan

2) Department of Internal Medicine, Kyushu University Beppu Hospital, 4546 Tsurumihara, Tsurumiji, Beppu, Oita, 874-0838, Japan

3) Toyama Chemical Co., Ltd., 4-1 Shimookui 2-chome, Toyama, 930-8508, Japan.

4) Department of Orthopaedic Surgery, Sainou Hospital, 70 Takata, Toyama 930-0866, Japan

5) Department of Anesthesiology, Faculty of Medicine, University of Toyama, 2630 Sugitani, Toyama 930-0194, Japan

6) Center for iPS Cell Research and Application, Kyoto University, 53 Kawahara-cho, Shogoin, Sakyo-ku, Kyoto 606-8507, Japan.

Corresponding author: Shoji Seki MD, PhD

Department of Orthopaedic Surgery, University of Toyama, Faculty of Medicine 2630 Sugitani, Toyama 930-0194, Toyama, Japan Tel: +81-76-434-7353 Fax: +81-76-434-5035

E-mail: [seki@med.u-toyama.ac.jp](mailto:seki@med.u-toyama.ac.jp)

**
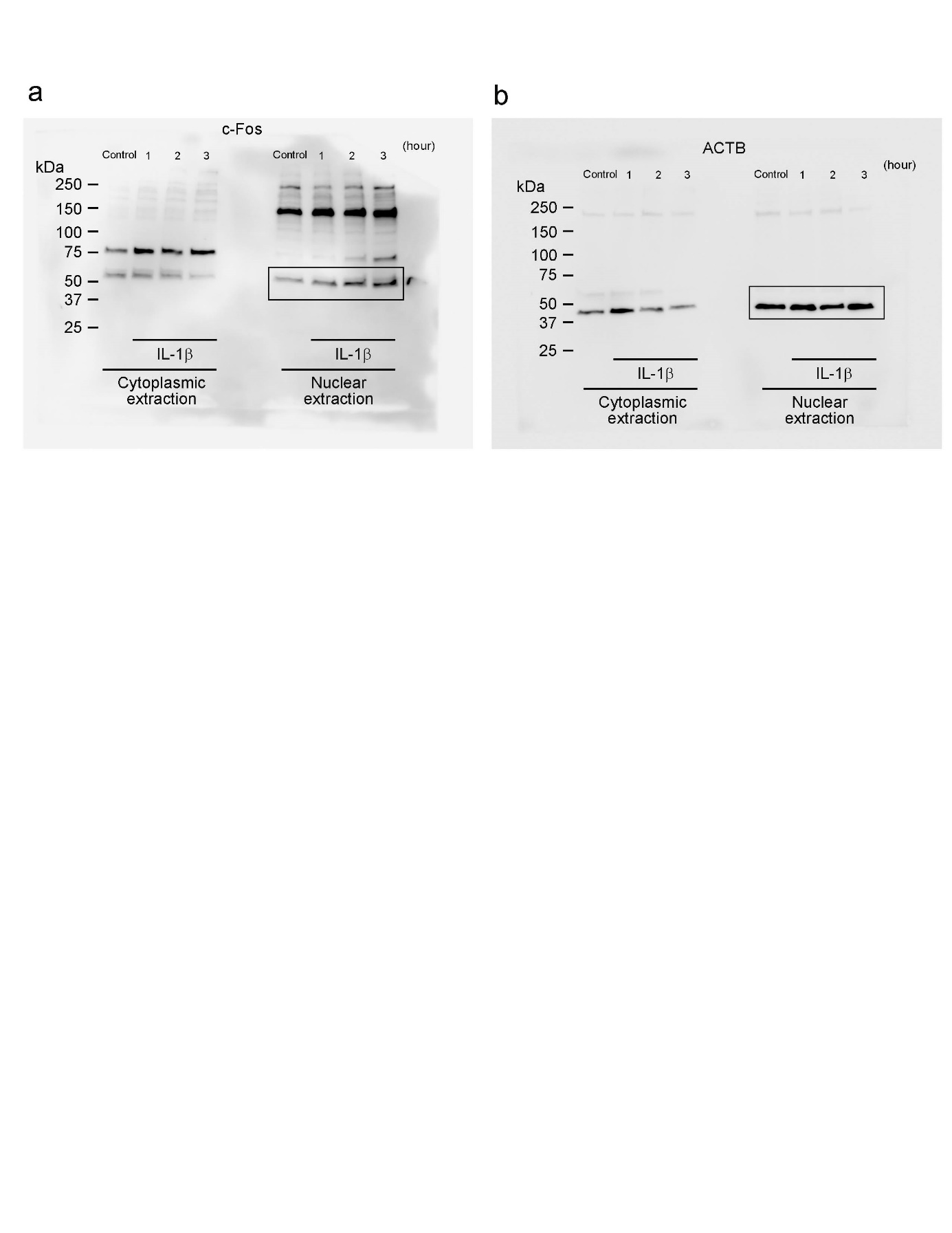
**

**Supplementary figure 1. Full scans of western bolts related to Figure 1b.**

**(a)** c-Fos**, (b)** ACTB. The square area is an image presented in Figure 1b.

**a b**

**
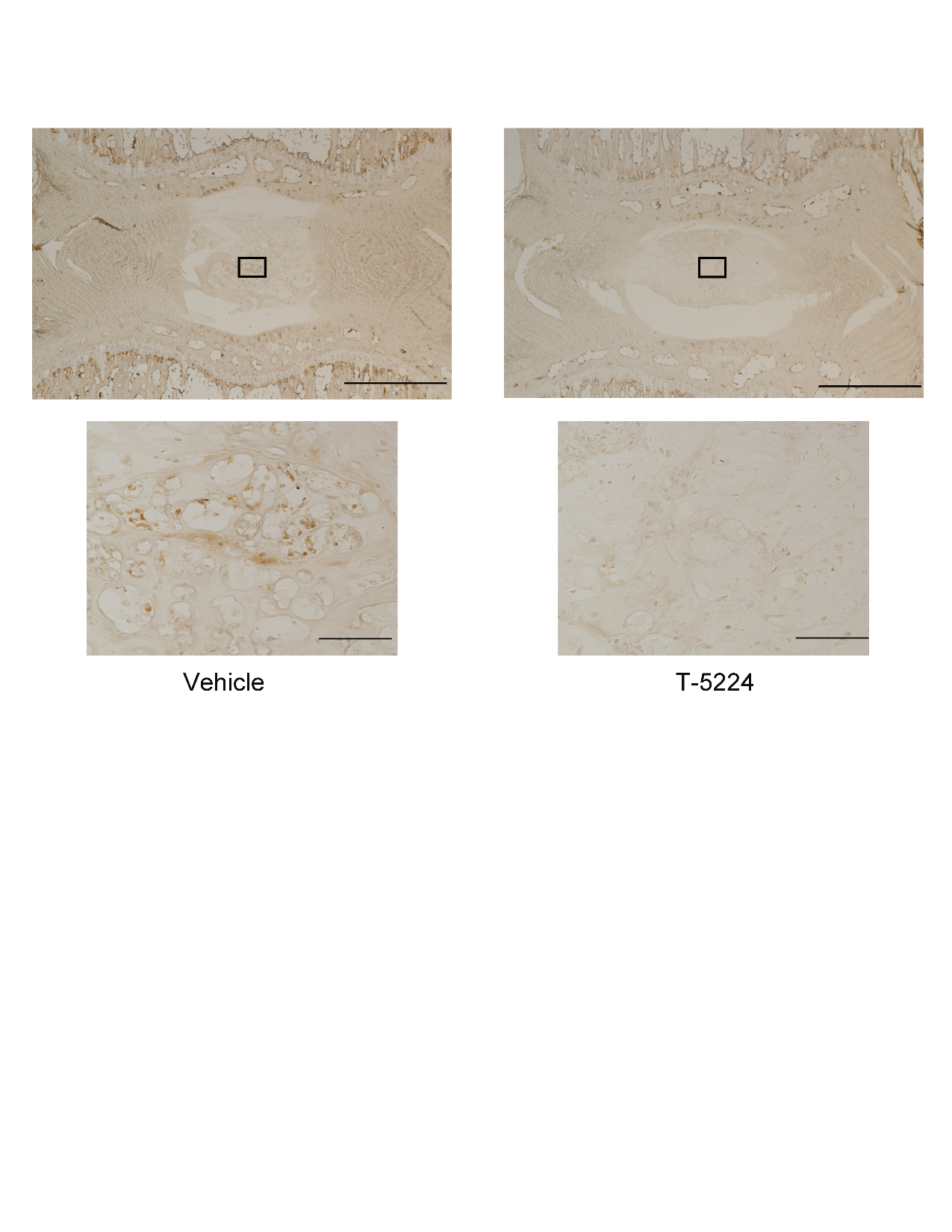
**

**Supplementary figure 2. Immunohistochemistry for MMP-13.**

Eight weeks after administration of (a) vehicle solution or (b) T-5224. Upper images show low magnification of the intervertebral disc, and lower images show higher magnification. Expression of MMP-13 was decreased in half-punctured IVD with T-5224 compared to that with vehicle. Scale bars = 1 mm in low magnification images, 100 μm in higher magnification images.

**Supplementary Table 1.** Primers used to amplify human genes.

|  |  |  |  |
| --- | --- | --- | --- |
|  | Primer | Sequence |  |
|  | *MMP-3* F | TGGCATTCAGTCCCTCTATGG |  |
|  | *MMP-3*R | AGGACAAAGCAGGATCACAGTT |  |
|  | *MMP-13* F | CAAAAACGCCAGACAAATGTGACC |  |
|  | *MMP-13* R | GATGCAGGCGCCAGAAGAATCT |  |
|  | *ADAMTS-5* F | GAACATCGACCAACTCTACTCCG |  |
|  | *ADAMTS-5* R | CAATGCCCACCGAACCATCT |  |
|  | *GAPDH* F | AATGGACAACTGGTCGTGGAC |  |
|  | *GAPDH* R | CCCTCCAGGGGATCTGTTTG |  |
|  | *c-Fos* F | CCGGGGATAGCCTCTCTTACT |  |
|  | *c-Fos* R | CCAGGTCCGTGCAGAAGTC |  |
|  | *IL-1β* F | AAACAGATGAAGTGCTCCTTCCAGG |  |
|  | *IL-1β* R | TGGAGAACACCACTTGTTGCTCCA |  |

**Supplementary Table 2.** Primers used to amplify mouse genes.

|  |  |  |  |
| --- | --- | --- | --- |
|  | Primer | Sequence |  |
|  | *Col2a1* F | TTGAGACAGCACGACGTGGAG |  |
|  | *Col2a1* R | AGCCAGGTTGCCATCGCCATA |  |
|  | *Mmp-3* F | GGCCTGGAACAGTCTTGGC |  |
|  | *Mmp-3* R | TGTCCATCGTTCATCATCGTCA |  |
|  | *Mmp-13* F | TGTTTGCAGAGCACTACTTGAA |  |
|  | *Mmp-13* R | CAGTCACCTCTAAGCCAAAGAAA |  |
|  | *Adamts-5* F | CCCAGGATAAAACCAGGCAG |  |
|  | *Adamts-5* R | CGGCCAAGGGTTGTAAATGG |  |
|  | *Gapdh* F | AAGCCCATCACCATCTTCCAGGAG |  |
|  | *Gapdh* R | ATGAGCCCTTCCACAATGCCAAAG |  |
|  | *c-Fos* F | CGGGTTTCAACGCCGACTA |  |
|  | *c-Fos* R | TTGGCACTAGAGACGGACAGA |  |
|  | *Il-1β* F | GAAATGCCACCTTTTGACAGTG |  |
|  | *Il-1β* R | TGGATGCTCTCATCAGGACAG |  |

**Supplementary Table 3.** Primers used to amplify rat genes.

|  |  |  |  |
| --- | --- | --- | --- |
|  | Primer | Sequence |  |
|  | *Gapdh* F | GGCACAGTCAAGGCTGAGAATG |  |
|  | *Gapdh* R | ATGGTGGTGAAGACGCCAGTA |  |
|  | *Mmp-3* F | TGATGGGCCTGGAATGGTC |  |
|  | *Mmp-3* R | TTCATGAGCAGCAACCAGGAATAG |  |
|  | *Mmp-13* F | CCCTGGAATTGGCGACAAAG |  |
|  | *Mmp-13* R | GCATGACTCTCACAATGCGATTAC |  |
|  | *Adamts-5* F | AGTGTCATACCCTGCCCACCTAAC |  |
|  | *Adamts-5* R | TGCGTATTTGGGAACCCATTCTA |  |
|  | *Calca* F | CCTGGTTGTCAGCATCTTGCTC |  |
|  | *Calca* R | TGCACCAGTGCAGCCAGTA |  |
|  | *Ngf* F | TGCCAAGGACGCAGCTTTC |  |
|  | *Ngf* R | TGAAGTTTAGTCCAGTGGGCTTCAG |  |
|  | *Pdyn* F | ACTGCCTGTCCTTGTGTTCC |  |
|  | *Pdyn* R | CCAAAGCAACCTCATTCTCC |  |
|  | *Penk* F | CGGCGACATCAACTTCCT |  |
|  | *Penk* R | CTCGGGCTTGGACACCT |  |
|  | *c-Fos* F | CGGGTTTCAACGCCGACTA |  |
|  | *c-Fos* R | TTGGCACTAGAGACGGACAGA |  |

**Supplementary Table 4.** Gene ontology analysis of genes down-regulated by T-5224 in the rat dorsal root ganglia.

| GOMFID | Term | P value |
| --- | --- | --- |
| GO:0001515 | [opioid peptide activity](http://amigo.geneontology.org/amigo/term/GO:0001515) | 0.0002 |
| GO:0031628 | [opioid receptor binding](http://amigo.geneontology.org/amigo/term/GO:0031628) | 0.0008 |
| GO:0019955 | [cytokine binding](http://amigo.geneontology.org/amigo/term/GO:0019955) | 0.0014 |
| GO:0016740 | [transferase activity](http://amigo.geneontology.org/amigo/term/GO:0016740) | 0.0039 |
| GO:0019838 | [growth factor binding](http://amigo.geneontology.org/amigo/term/GO:0019838) | 0.0042 |
| GO:0004919 | [interleukin-9 receptor activity](http://amigo.geneontology.org/amigo/term/GO:0004919) | 0.0048 |
| GO:0004584 | [dolichyl-phosphate-mannose-glycolipid alpha-mannosyltransferase activity](http://amigo.geneontology.org/amigo/term/GO:0004584) | 0.0048 |
| GO:0019983 | [interleukin-9 binding](http://amigo.geneontology.org/amigo/term/GO:0019983) | 0.0048 |
| GO:0016301 | [kinase activity](http://amigo.geneontology.org/amigo/term/GO:0016301) | 0.0048 |
| GO:0030291 | [protein serine/threonine kinase inhibitor activity](http://amigo.geneontology.org/amigo/term/GO:0030291) | 0.0068 |
| GO:0005184 | [neuropeptide hormone activity](http://amigo.geneontology.org/amigo/term/GO:0005184) | 0.0090 |
| GO:0004798 | [thymidylate kinase activity](http://amigo.geneontology.org/amigo/term/GO:0004798) | 0.0095 |
| GO:0004911 | [interleukin-2 receptor activity](http://amigo.geneontology.org/amigo/term/GO:0004911) | 0.0095 |
| GO:0016155 | [formyltetrahydrofolate dehydrogenase activity](http://amigo.geneontology.org/amigo/term/GO:0016155) | 0.0095 |
| GO:0016316 | [phosphatidylinositol-3,4-bisphosphate 4-phosphatase activity](http://amigo.geneontology.org/amigo/term/GO:0016316) | 0.0095 |
| GO:0080019 | [fatty-acyl-CoA reductase (alcohol-forming) activity](http://amigo.geneontology.org/amigo/term/GO:0080019) | 0.0095 |
| GO:0050062 | [long-chain-fatty-acyl-CoA reductase activity](http://amigo.geneontology.org/amigo/term/GO:0050062) | 0.0095 |
| GO:0005130 | [granulocyte colony-stimulating factor receptor binding](http://amigo.geneontology.org/amigo/term/GO:0005130) | 0.0095 |
| GO:0032089 | [NACHT domain binding](http://amigo.geneontology.org/amigo/term/GO:0032089) | 0.0095 |
